# Influence of Polarity Arrangement of Inter-Wire Arc on Droplet Transfer in Cross-Coupling Arc Welding

**DOI:** 10.3390/ma12233985

**Published:** 2019-12-01

**Authors:** Shanwen Dong, Fan Jiang, Bin Xu, Shujun Chen

**Affiliations:** 1 Engineering Research Center of Advanced Manufacturing Technology for Automotive Components, Ministry of Education, Beijing University of Technology, Beijing 100124, China; D_shanwen@163.com (S.D.); cougarxbin@163.com (B.X.); sjchen@bjut.edu.cn (S.C.); 2 Beijing Engineering Researching Center of laser Technology, Beijing University of Technology, Beijing 100124, China

**Keywords:** cross-coupling arc, inter-wire arc (IWA), polarity arrangement, droplet transfer

## Abstract

In order to reduce the influence of polar zone effect in cross-coupling arc by changing inter-wire arc (IWA) configuration, the influence of polarity arrangement of the IWA on droplet transfer was studied. The change of voltage-current waveform and the process of droplet transfer were recorded and analyzed by a high-speed camera and electric signal synchronous acquisition system. The results show that when the IWA polarity is arranged as anode on the bottom and cathode on top, the anode spot force always promotes the droplet transfer and reduces the critical current value of spray transfer. However, with the increase in the input voltage of the IWA, the resistance of the cathode spot force becomes obvious, which hinders the droplet transfer. While the IWA polarity is arranged as anode on top and cathode on the bottom, increasing the input voltage of the IWA obviously reduces the plasma arc voltage. The critical current of spray transfer increases in anode droplet, while the cathode droplet is mainly globular transfer, and there is no spatter explosion process. Through a comprehensive comparison of the droplet transfer process of anode and cathode under the different IWA polarity arrangement, the process of anode and cathode in the IWA polarity arrangement of the anode on top and the cathode on the bottom is more stable than that in the IWA polarity arrangement of the anode on the bottom and the cathode on top, mainly because the cathode spot force under high current do not hinder the cathode droplet.

## 1. Introduction

Due to the high welding efficiency and good welding quality, gas metal arc welding (GMAW) is widely used in aerospace, shipbuilding, vehicle engineering, and other industries [1,2,3,4,5]. However, the thermal coupling coefficient of a single consumable electrode arc is large. If the welding current is increased to further improve the welding deposition rate, at the same time, the thermal input to the molten pool or the workpiece will inevitably increase, resulting in uncontrollable welding quality. Therefore, a variety of hybrid welding processes have been proposed so far, which are mainly divided into two categories: One is the hybrid welding with the workpiece as the coupling carrier, such as twin-wire tandem welding [6,7,8], tungsten inert gas-metal inert gas (TIG-MIG) welding [9,10], plasma-MIG welding [11,12], which improves the deposition rate by means of parallel multi-wire arcs forming or preheating wire, but the workpiece will also be forced to accept too much heat input. The second is the hybrid welding with the welding wire or tungsten electrode as the coupling carrier, such as double electrode gas metal arc welding [13,14,15], arcing-wire PAW (plasma arc welding) [16,17], double wire dynamic three arc welding [18], which improves the wire deposition rate through the bypass electrode shunting the main arc current, and the workpiece is only one electrode, reducing the thermal transmission of the coupling arc to it. However, it is necessary to increase the length of the main arc to avoid the interference of the bypass arc on the main arc when it is coupled, and only under a certain main arc current can stable droplet transfer be achieved, which has certain restrictions on the application of low heat input welding [19].

As a new welding process in recent years, cross-coupling arc makes a non-consumable electrode arc (called main arc) and a consumable electrode arc (called inter-wire arc) forcibly cross-coupled in a limited space. The main arc burns between the workpiece and the tungsten electrode, which is mainly responsible for the thermal input of the workpiece, while the inter-wire arc (IWA) forms an arc between two welding wires, which is mainly responsible for metal deposition, and there is no electrical connection with the workpiece. The parameters of the main arc and the IWA can be adjusted respectively to improve the wire deposition rate effectively without changing the thermal input to the weld pool.

**Figure 1:**
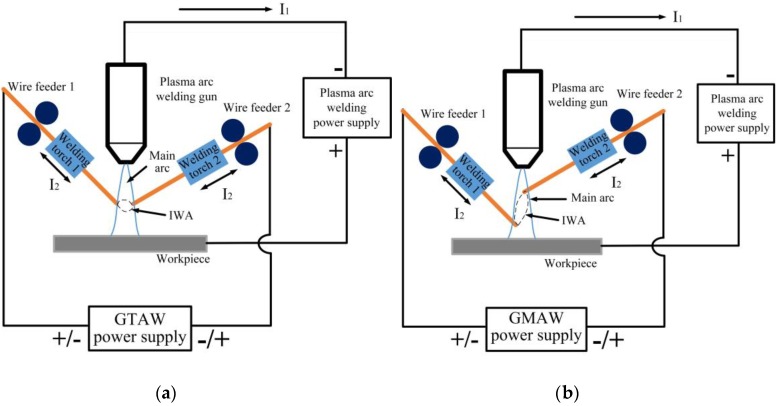
Schematic diagram of the modified cross-coupling arc welding. (**a**) Initial design; (**b**) improved and optimized design.

At present, the research on cross-coupling arc welding mainly focuses on arc stability, arc behavior, and droplet transfer [20,21,22,23,24], especially arc stability tests playing a primary role in the development of welding processes, which is an important mean to determine whether the arc can perform effective thermal output and whether the droplet transition is stable [25,26]. However, it is found that there is an obvious polar zone phenomenon in the study of cross-coupling arc stability, that is, the main arc is regularly inclined to the polar zone of the IWA. The existence of the polar phenomenon not only affects the stability of the main arc, but also reduces the arc force of the main arc on the workpiece, resulting in shallow weld penetration. The polar zone phenomenon is due to the horizontal component of the magnetic field produced by the IWA, and the macroscopic performance is the effect of Ampere force. For this reason, this paper proposes to change the IWA configuration in order to adjust the current direction in the IWA and change its magnetic field distribution. As shown in Figure 1, the two welding wires are distributed up and down on both sides of the main arc center. At the same time, GMAW power supply with constant voltage characteristic is used instead of GTAW power supply with constant current characteristic to increase the regulating effect of the IWA itself. Due to the up and down distribution of the two welding wires, the difference of the IWA polarity arrangement will inevitably lead to the different welding current direction, and the welding current directly determines the arc thermal output, which affects the cross-coupling arc characteristics and droplet transfer process. In this paper, the droplet transfer process of cathode and anode under the different IWA polarity arrangement is studied, and the influence of the IWA polarity arrangement on the main arc voltage, the IWA current, and wire feed speed of cathode and anode is compared and analyzed, in order to establish a theoretical foundation for the application and optimization of the cross-coupling arc welding process.

## 2. Experimental Procedure

### 2.1. Experimental Materials and System

**Figure 2:**
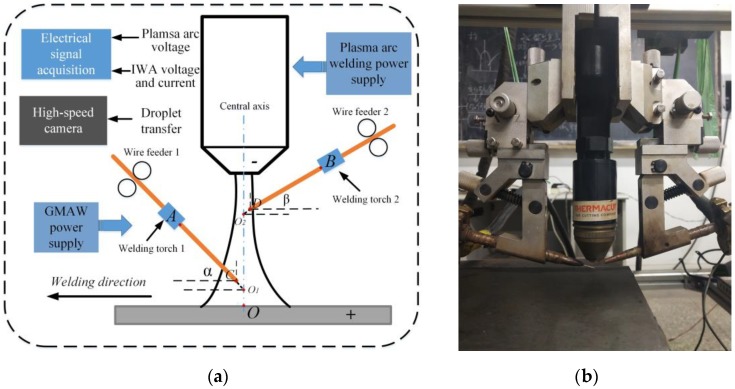
Schematic diagram of experimental system and physical drawing of welding gun. (**a**) Experiment system; (**b**) physical drawing of welding gun.

As shown in Figure 2, the experimental system mainly included two parts: Welding system and high-speed camera and the electrical signal synchronous acquisition system. The welding system consisted of a plasma arc welding power supply with constant current characteristic developed by Beijing University of Technology, an Aotai welding machine and two independent wire feeders. The plasma arc welding power supply provided the plasma main arc, Aotai welding machine was used to generate the IWA, and two wire feeders were independent of the welding machine, which respectively provided stable wire feed speed for two welding wires. The electric signal system collected the waveform of main arc voltage and IWA current in real time, and recorded the droplet transition process in real time with a high-speed camera. The parameters of the corresponding experimental setup are shown in Table 1.

### 2.2. Experimental Design

Due to the constant voltage characteristic of the IWA power supply, its input voltage mainly determines the thermal output of the IWA and affects the droplet transition behavior. Therefore, only the IWA polarity arrangement and input voltage of the IWA were changed during the experiment, as shown in Table 2. The effect of the IWA input voltage on the droplet transfer behavior of the anode and cathode under different IWA polarity arrangements was studied. Other welding conditions remained the same, in which O_1_ and O_2_ were the intersection points of the wire center extension line and central axis of the main arc, respectively.

## 3. Results and Discussion

### 3.1. The Droplet Transfer Behavior of the IWA with Anode on the Bottom and Cathode on the Top

**Figure 3:**
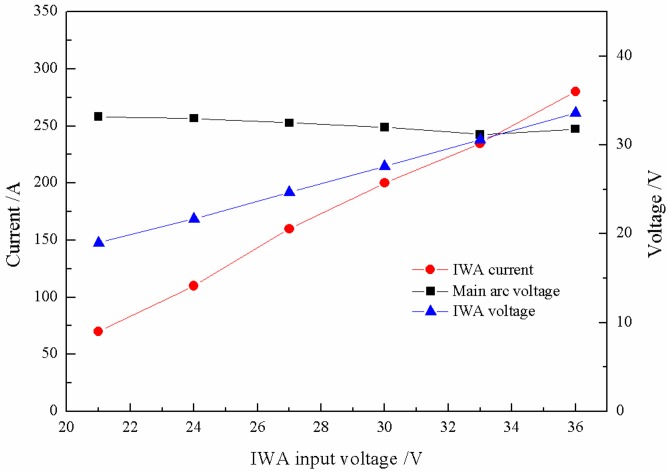
Influence of inter-wire arc (IWA) input voltage on the main arc voltage and IWA current.

Under the condition of the main arc constant current and the IWA constant voltage output, the droplet transfer process directly depends on the thermal input of cross-coupling arc, so it is necessary to understand the changes of the main arc voltage and the IWA current output. As shown in Figure 3, when the IWA polarity is arranged with anode on the bottom and cathode on the top, with the increase of the input voltage, the difference between the actual voltage of the IWA and the input voltage is 2 to 2.5 V, which mainly falls on the extension length of welding wire; the IWA current increases approximately linearly, and the current increases 35 to 45 A for every 3 V increase of the input voltage; while the main arc voltage decreases slowly and remains at about 32.5 V, it can be considered that the main arc voltage has no significant effect. When the input voltage of the IWA increases, the heat generation power of the arc column increases obviously, while the IWA arc column and the main arc column are closely coupled, sharing the same gas conductive channel, and the temperature of the main arc column increases correspondingly. On the one hand, the resistivity of the main arc column decreases, on the other hand, the number of charged particles in the IWA arc column increases, and they flow through the conductive channel at the same time, which hinders the movement of the charged particles in the original main arc, leading to the main arc impedance basically unchanged, so the main arc voltage is basically the same.

**Figure 4:**
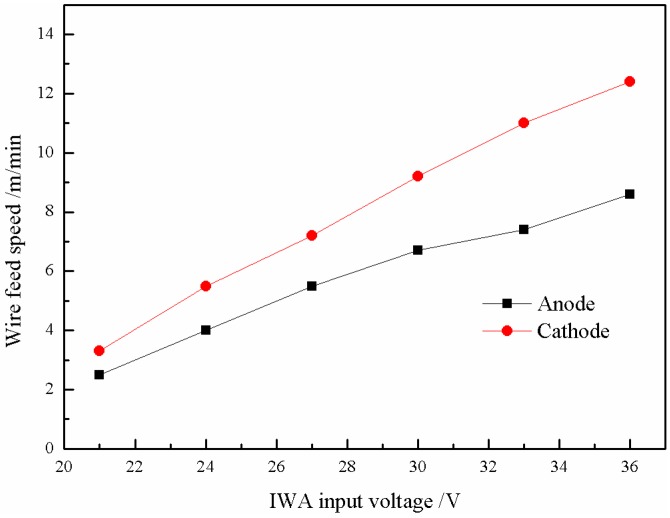
Change trend of wire feed speed with IWA input voltage.

The setting criteria of wire feed speed should first ensure that the transfer of cathode and anode droplets is carried out on both sides of the centerline of the main arc, respectively, and cannot interfere with each other’s transition, and the second is the transfer process within the main arc. The melting heat of the wires comes from the IWA itself and the main arc column. The melting speed can be adjusted by using the radial temperature gradient of the main arc column and self-regulation of the IWA, and the melting speed can be adjusted in a wide range. Generally, as long as the wire feed speed is not too large, which causes the wire to cross the central line of the main arc and insert into the molten pool, there is always a suitable melting speed corresponding to it. In this paper, the wire feed speed is obtained by a large number of experiments on the basis of the criteria. Figure 4 shows the relationship between the IWA input voltage and wire feed speed of anode and cathode. It can be seen that with the increase of the IWA input voltage, the wire feed speed of anode and cathode basically increases linearly. When the input voltage is 36 V, the wire feed speed of anode and cathode respectively reaches 12.5 and 8.1 m/min. When the input voltage is greater than 30 V, the welding current is further increased, and the electromagnetic shrinkage force is significantly enhanced, which results in the end of the welding wire being thinned, the current density is sharply increased, and the anode voltage drop is reduced, so the rising slope of the anode wire feed speed is decreased, and the difference between the anode and the cathode wire feed speed is gradually increased.

**Figure 5:**
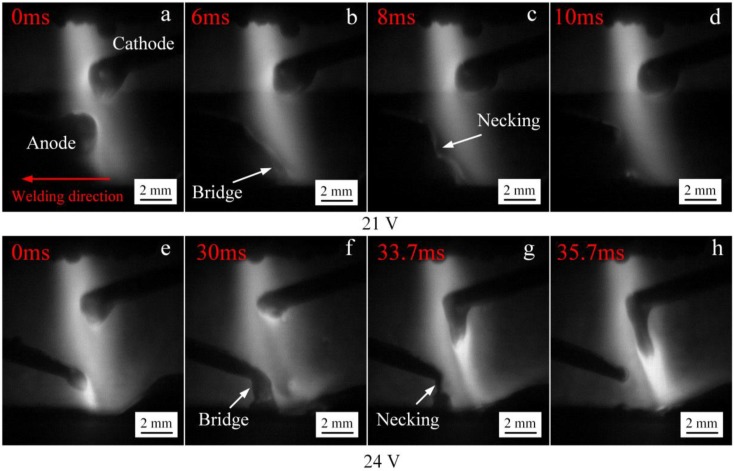
The process of anode droplet transfer at different IWA input voltages. (**a**–**d**) 21 V; (**e**–**h**) 24 V.

Figure 5 shows the process of anode droplet transfer with anode below cathode at low input voltage of the IWA. As a result of the low input voltage of the IWA, the welding current is relatively small. On the one hand, the heat production power of the IWA anode area is small, the melting speed of the welding wire is slow, and the transfer frequency of droplet is low. On the other hand, the IWA arc force does not play a major role, relying on the gravity of the droplet itself and the plasma flow force of main arc to promote the droplet transfer. It can be seen from Figure 5a,e that when the input voltage is 21 V and the input voltage is 24 V, the size of the anode droplet is significantly reduced, because the increase of the input voltage and the temperature of the droplet make the surface tension coefficient decrease. As the anode welding wire is close to the workpiece end, the bottom of the droplet in Figure 5b,f contacts the surface of the molten pool to form a liquid bridge. At this time, the surface tension effect of the molten pool on the droplet appears, and the droplet is elongated, but the top of the droplet has not yet separated from the end of the welding wire. The necking phenomenon appears between the droplet and the welding wire in Figure 5c,g, but there is no rupture and splash, indicating that there is no current flowing through the necking section at this time. The droplet transition frequencies in Figure 5d,h are 10 and 24 Hz, respectively.

**Figure 6:**
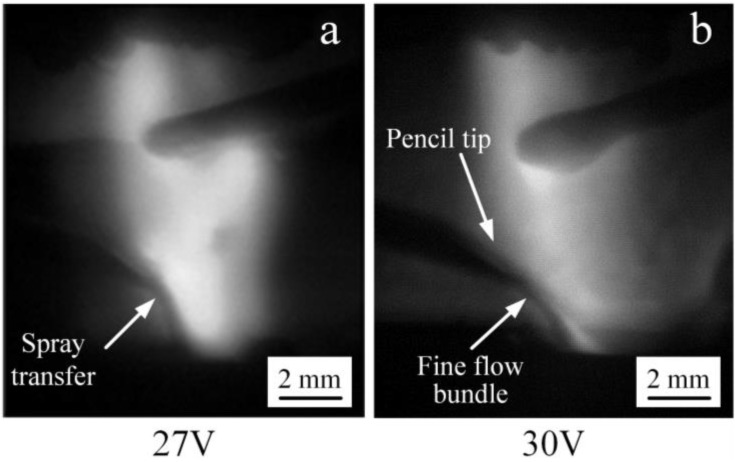
The process of anode droplet transfer at different IWA input voltages. (**a**) 27 V; (**b**) 30 V.

When the input voltage of the IWA is further increased to 27 and 30 V, the anode droplet transfer process is shown in Figure 6. At this time, the IWA current is 160 and 200 A, the resistance heat of the anode wire end and the IWA power are significantly increased, the melting speed of the welding wire is accelerated, and the welding wire end is thinned under the shear effect of electromagnetic force. In Figure 6a,b, the droplet and jet form appear, respectively. When the input voltage is 30 V, the arc root jumps to the necking root, and the anode spot force mainly promotes the droplet transfer, and the droplet basically forms a fine flow column along the axis of the welding wire. Compared with conventional MAG/MIG welding, the critical current values of spray transfer and streaming transfer are obviously reduced. When the input voltage of the IWA is increased to 33 and 36 V, the IWA current is 235 and 280 A, the effect of electromagnetic contraction force on arc heat and resistance heat is more obvious, and the anode droplet is easier to achieve streaming transfer.

**Figure 7:**
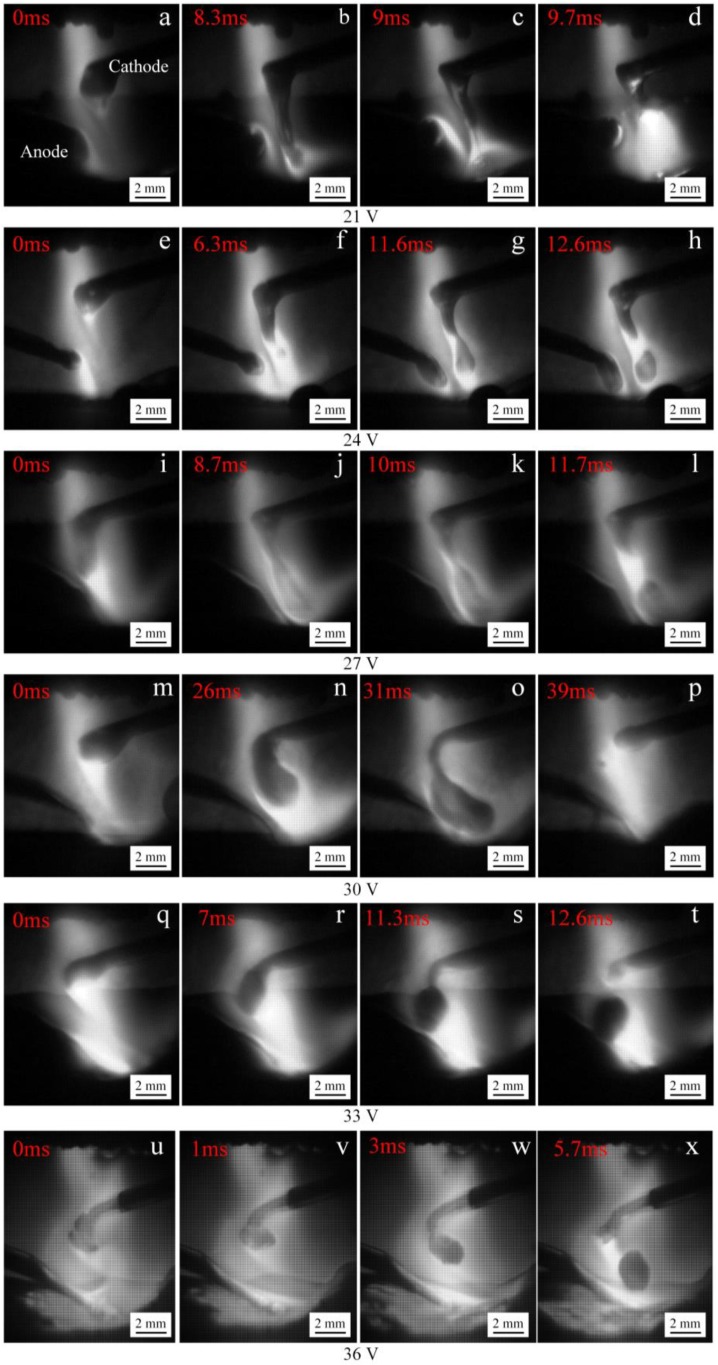
The process of cathode droplet transfer at different IWA input voltages. (**a**–**d**) 21 V; (**e**–**h**) 24 V; (**i**–**l**) 27 V; (**m**–**p**) 30 V; (**q**–**t**) 33 V; (**u**–**x**) 36 V.

Figure 7 shows the process of cathode droplet transfer under different IWA input voltages. When the input voltage is 21 V, the heat input at the welding wire end is insufficient, the IWA arc force does not play the main role, the metal liquid is elongated into a long liquid flow column, and the bottom of the liquid flow column contacts the surface of the molten pool under the action of its own gravity and the main arc plasma flow force, forming a necking at the bottom of the liquid flow column, because the necking place is broken at this time, indicating that there is a current passing through. When the input voltage is 24, 27, and 30 V, it can be seen that the droplet diameter increases significantly. Although the increase of the IWA current increases the resistance heat of cathode wire and the heat production power of cathode area, and the end of wire becomes thinner, at this time, the cathode spot is at the bottom of the droplet. The larger the welding current is, the more the negative spot force formed hinders the droplet transition upward until the arc root jumps to the necking point, and the droplet is the larger drop form transition. When the input voltage is 33 V, the droplet diameter is obviously reduced. At this time, the IWA current is 235 A, the cathode spot is at the end of the welding wire, and the spot area is expanded. The cathode spot moves from the bottom of the liquid to the side, which reduces the obstruction to the droplet transition. The droplet shape becomes a sphere, and the necking and falling off are formed between the welding wire end and the droplet. When the input voltage is 36 V, a small liquid flow column is formed at the welding wire end, and there is a certain repulsion effect. The droplet does not always fall off along the center of the main arc, but inclines to the right side of the main arc, and the cathode droplet presents a spray droplet transfer state, and the transfer frequency increases obviously.

### 3.2. The Droplet Transfer Behavior of the IWA with Anode on the Top and Cathode on the Bottom

**Figure 8:**
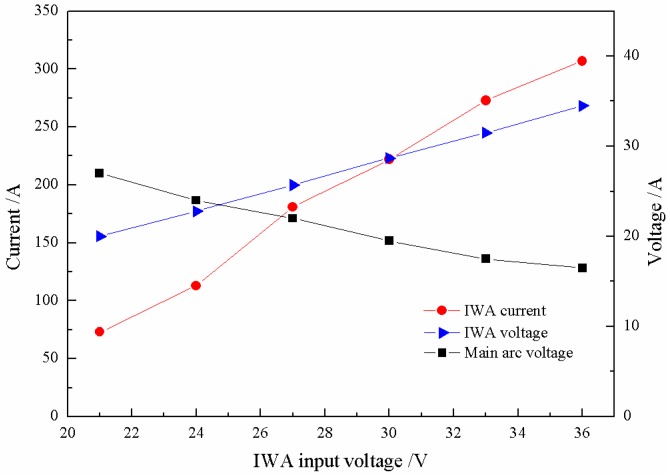
The variation trend of the main arc voltage and IWA current with IWA input voltage.

Figure 8 shows the relationship between the IWA input voltage, the IWA current, and main arc voltage. With the increase of input voltage, the difference between the IWA actual voltage and input voltage is 1 to 1.5 V; when the output voltage is greater than 27 V, the IWA current basically increases linearly. Compared with Figure 3, the IWA current is 20 to 35 A higher under the same input voltage; the biggest difference is the degree of decrease of the main arc voltage.

When the polarity arrangement of the IWA is anode on top and cathode on the bottom, the IWA arc column area and the main arc column area are closely coupled and included in the main arc. In order to meet the principle of minimum energy consumption, the number of ions supplied from the IWA anode area to the arc column area will be partially selected to move directly to the main arc cathode area, and the electrons supplied from the corresponding main arc cathode area to the arc column area will also be partially selected to move to the IWA anode area, thus reducing the movement path of cations and electrons, and the resistance encountered will also be correspondingly reduced. Similarly, the electrons provided by the IWA cathode area and the cations provided by the anode area of the main arc are partially transferred to the anode area of the main arc and the cathode area of the IWA. Therefore, the impedance of the IWA arc column and that of the main arc column decrease relatively at the same time. Compared with the IWA polarity arrangement with anode below cathode, the IWA current increases and the main arc voltage decreases under the same input voltage of the IWA.

**Figure 9:**
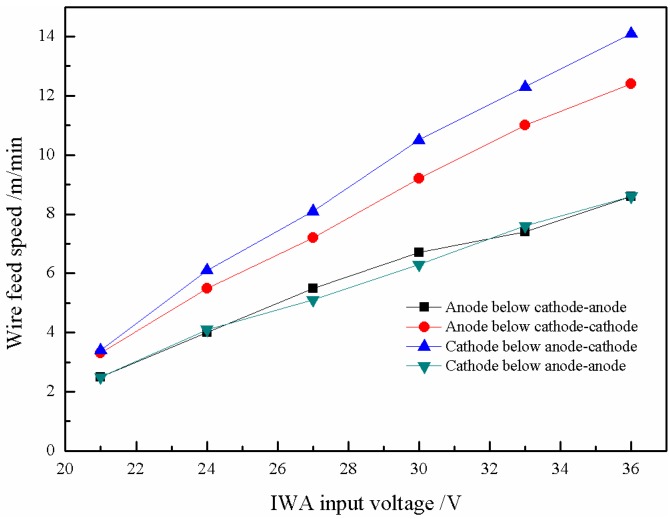
Relationship between the wire feed speed and IWA input voltage under different IWA polarity arrangements.

Based on the same criteria of wire feed speed, the wire feed speed is also obtained when the polarity arrangement of the IWA is anode on top and cathode on the bottom. Figure 9 shows the influence of the IWA input voltage on the wire feed speed of cathode and anode. It can be seen that when the IWA polarity is arranged with cathode below anode, the wire feed speed of cathode and anode basically increases linearly with the increase of the IWA input voltage. When the input voltage is 36 V, the increment of cathode wire feed speed is 1.8 m/min. Due to the distribution of charged particles movement caused by the polarity arrangement, the welding current increases and the cathode wire feed speed increases. For the anode welding wire, the anode spot force plays a certain role. As a result, there is no obvious difference in wire feed speed between the anode wires.

**Figure 10:**
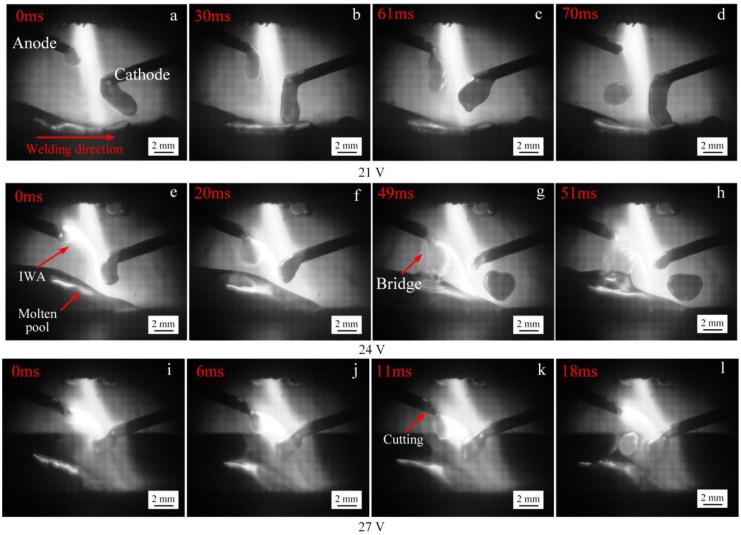
The transfer process of anode droplet at IWA input voltages. (**a**–**d**) 21 V; (**e**–**h**) 24 V; (**i**–**l**) 27 V.

Figure 10 shows the process of anode droplet with cathode below anode at low input voltage of the IWA. When the input voltage is 21, 24, and 27 V, it can be seen that the size of anode droplet decreases gradually. On the one hand, with the increase of heat production in anode area and resistance at the end of welding wire, the increase of droplet temperature leads to the decrease of surface tension coefficient, which falls off under the action of self gravity and main arc plasma flow, on the other hand, the end of anode welding wire is electrified under the action of the magnetic shrinkage force, the surface tension of the welding wire to the droplet is reduced, the droplet diameter becomes smaller, the droplet transfers from big drop to fine drop along the bottom of the welding wire end, and the droplet transfer frequency is accelerated.

**Figure 11:**
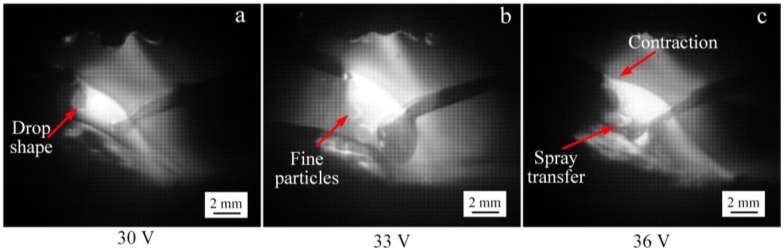
The droplet transfer process of anode at different IWA input voltages. (**a**) 30 V; (**b**) 33 V; (**c**) 36 V.

Further increasing the input voltage of the IWA, and the process diagram of anode droplet transfer is shown in Figure 11. It can be seen that when the input voltage is 30 V, the IWA current is 220 A, the droplet diameter is approximately equal to the diameter of the welding wire, and the droplet transition is mainly to the molten pool. When the input voltage is 33 V and the IWA current is 270 A, the IWA electromagnetic contraction force is obviously enhanced, the area of the anode arc root is contracted, and the droplet is transferred into the molten pool as a droplet, the droplet diameter is obviously smaller than the wire diameter, and the droplet transfer frequency is about 100 Hz. When the output voltage is 36 V, the IWA current is 307 A, the part near the IWA arc column shrinks further, and the droplet transits into the molten pool in the form of a jet in the IWA arc column. Compared with the IWA polarity arrangement with anode below cathode, the critical current value of the spray droplet transfer of the anode increases at the same IWA voltage. The difference of the IWA current is about 110 A, which is mainly due to the effect of the anode spot force on the droplet transfer.

**Figure 12:**
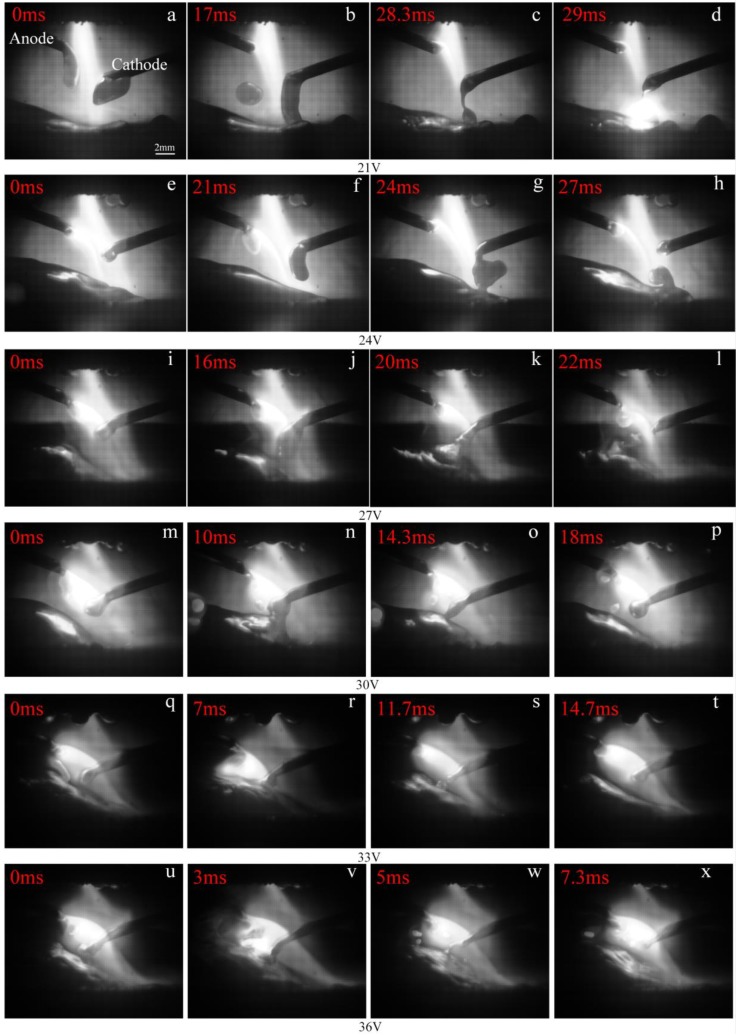
The process of cathode droplet transfer under different IWA input voltages. (**a**–**d**) 21 V; (**e**–**h**) 24 V; (**i**–**l**) 27 V; (**m**–**p**) 30 V; (**q**–**t**) 33 V; (**u**–**x**) 36 V.

Figure 12 shows the process of cathode droplet when the polarity arrangement of IWA is anode on top and cathode on the bottom. With the increasing input voltage of the IWA, the droplet transition has a bridging transition, a large droplet transition and a small droplet transition, and there is no droplet transition. When the input voltage of IWA is 21 and 24 V, the welding current is 70 and 113 A, and the thermal output of IWA is relatively insufficient. Under the action of gravity and plasma flow force, the bottom of the droplet contacts with the surface of the molten pool to form a liquid bridge, and there is necking between the droplet and the welding wire, without explosion. The droplet transition is bridging and large droplet transition, respectively. When the input voltage of IWA is increased to 27, 30, 33, and 36 V, respectively, the maximum value of IWA current is 300 A. As the melting speed of cathode wire increases, but the welding speed remains unchanged during the test, the distance from the top of weld pool to cathode wire shortens, and the bottom of droplet also contacts with the surface of weld pool to form a liquid bridge. The surface tension of weld pool plays a role of lubrication and pulling on the droplet transition. The transition frequency is accelerated and there is no splash phenomenon. If the welding speed is suitable, the cathode droplet presents a droplet transition, and the droplet diameter is about equal to the wire diameter. Compared with the polar arrangement of IWA with the anode on the lower cathode, the droplet transfer process of the cathode is stable, and there is no droplet rejection phenomenon. It needs more IWA current to realize the spray transfer.

Through the above analysis and comparison, when the polarity arrangement of the IWA is the anode on the bottom and the cathode on top, with the increase of the IWA input voltage, the effect of anode droplet spot force is prominent, reducing the critical current value of the spray droplet, and it is easy to realize spray transfer and streaming transfer, but the cathode spot force hinders the formation of large droplet transition and repulsive transition, so it is suitable for the occasions with small IWA parameters. While the polarity arrangement of the IWA is that the anode is at the top and the cathode is at the bottom, as the input voltage of the IWA increases, the spot force of the anode plays a certain role in hindering and increasing the critical current value of the spray transfer, but at this time, the spot force of the cathode does not hinder. The droplet bridging transition is the main part, and the welding process is stable, which is more suitable for the situation with larger IWA parameters.

## 4. Conclusions

In this study, the existing cross-coupling arc welding was modified by adjusting the inter-wire arc (IWA) configuration and the influence of the IWA polarity arrangement on the droplet transfer process was investigated experimentally. The following conclusions can be drawn:

(1) Under the same input voltage of the IWA, the main arc voltage with anode on bottom and cathode on top is higher than that with anode on top and cathode on the bottom, while the IWA current is the opposite.

(2) The anode spot force promotes droplet transfer and reduces the critical current of spray transfer with the IWA polarity arrangement of anode on the bottom and cathode on top. However, for the cathode droplet, increasing the IWA input voltage, the effect of cathode spot force becomes obvious, which hinders the droplet transfer.

(3) Increasing the IWA input voltage significantly reduces the plasma main arc voltage with the IWA polarity arrangement of anode on top and cathode on the bottom. The anode droplet has no streaming transfer, while the cathode droplet is mainly globular transfer, without the spatter explosion process.

## Figures and Tables

**Table 1:** Technical specifics of the experimental setup.

Equipment and Accessories	Model, Materials, or Size
Plasma arc welding power supply	PLASMA-300
GMAW power supply	MAG-350RPL
Tungsten setback	4 mm
Diameter of nozzle	3.2 mm
Diameter of tungsten	5 mm
High-speed camera	IDT motion Y4
Frame number	3000 f/s
Ion gas	Argon gas (purity 99.9%)
Shielding gas	A mixture of argon gas (purity 80%) and carbon dioxide gas (purity 20%)
Base metal	Q345 steel
Dimension of base metal	200 × 150 × 8 mm
Welding wire	ER70S-6
Diameter of welding wire	1.2 mm

**Table 2:** Experimental parameters.

**Number**	**Main Arc Current** **(A)**	**Nozzle Height** **(mm)**	**Shielding Gas Flow Rate** **(L/min)**	**Plasma Gas Flow Rate** **(L/min)**	**Welding Speed** **(mm/s)**
1-1	100	10	15	2.0	5
1-2	100	10	15	2.0	5
1-3	100	10	15	2.0	5
1-4	100	10	15	2.0	5
1-5	100	10	15	2.0	5
1-6	100	10	15	2.0	5
2-1	100	10	15	2.0	5
2-2	100	10	15	2.0	5
2-3	100	10	15	2.0	5
2-4	100	10	15	2.0	5
2-5	100	10	15	2.0	5
2-6	100	10	15	2.0	5
**Number**	**Wire Extension Length** **(mm)**	**Wire Angle** **α and β** **(°)**	**Wire Height** **OO_1_ and OO_2_** **(mm)**	**IWA Polarity Arrangement**	**IWA Input Voltage** **(V)**
1-1	10	25:30	3:7	Anode below cathode	21
1-2	10	25:30	3:7	Anode below cathode	24
1-3	10	25:30	3:7	Anode below cathode	27
1-4	10	25:30	3:7	Anode below cathode	30
1-5	10	25:30	3:7	Anode below cathode	33
1-6	10	25:30	3:7	Anode below cathode	36
2-1	10	25:30	7:3	Cathode below anode	21
2-2	10	25:30	7:3	Cathode below anode	24
2-3	10	25:30	7:3	Cathode below anode	27
2-4	10	25:30	7:3	Cathode below anode	30
2-5	10	25:30	7:3	Cathode below anode	33
2-6	10	25:30	7:3	Cathode below anode	36
